# Anti-Inflammatory Activity and ROS Regulation Effect of Sinapaldehyde in LPS-Stimulated RAW 264.7 Macrophages

**DOI:** 10.3390/molecules25184089

**Published:** 2020-09-07

**Authors:** Seung-Hwa Baek, Tamina Park, Myung-Gyun Kang, Daeui Park

**Affiliations:** 1 Department of Predictive Toxicology, Korea Institute of Toxicology, Daejeon 34114, Korea; seunghwa.beak@kitox.re.kr (S.-H.B.); tamina.park@kitox.re.kr (T.P.); myung-gyun.kang@kitox.re.kr (M.-G.K.); 2 Center for Convergent Research of Emerging Virus Infection, Korea Research Institute of Chemical Technology, Daejeon 34114, Korea; 3 Department of Human and Environmental Toxicology, University of Science and Technology, Daejeon 34113, Korea

**Keywords:** sinapaldehyde, anti-inflammatory effect, nitric oxide, reactive oxygen species, cytokine, docking simulation

## Abstract

We evaluated the anti-inflammatory effects of SNAH in lipopolysaccharide (LPS)-stimulated RAW 264.7 macrophages by performing nitric oxide (NO) assays, cytokine enzyme-linked immunosorbent assays, Western blotting, and real-time reverse transcription-polymerase chain reaction analysis. SNAH inhibited the production of NO (nitric oxide), reactive oxygen species (ROS), tumor necrosis factor (TNF)-α, and interleukin (IL)-6. Additionally, 100 μM SNAH significantly inhibited total NO and ROS inhibitory activity by 93% (*p* < 0.001) and 34% (*p* < 0.05), respectively. Protein expression of inducible nitric oxide synthase (iNOS) and cyclooxygenase-2 (COX-2) stimulated by LPS were also decreased by SNAH. Moreover, SNAH significantly (*p* < 0.001) downregulated the TNF-α, IL-6, and iNOS mRNA expression upon LPS stimulation. In addition, 3–100 µM SNAH was not cytotoxic. Docking simulations and enzyme inhibitory assays with COX-2 revealed binding scores of −6.4 kcal/mol (IC_50_ = 47.8 μM) with SNAH compared to −11.1 kcal/mol (IC_50_ = 0.45 μM) with celecoxib, a known selective COX-2 inhibitor. Our results demonstrate that SNAH exerts anti-inflammatory effects via suppression of ROS and NO by COX-2 inhibition. Thus, SNAH may be useful as a pharmacological agent for treating inflammation-related diseases.

## 1. Introduction

Inflammatory reactions typically occur as a natural physiological response against injurious stimuli such as the infectious invasion of pathogens and toxins [1]. However, uncontrolled and aberrant inflammation leads to many diseases such as rheumatoid arthritis [2], atherosclerosis [3], asthma [4], diabetes [5], chronic hepatitis [6], septic shock [7], and inflammatory neurodegenerative diseases [8]. During chronic inflammation, activated macrophages secrete high chemokines (interleukin-8 [IL-8], CC-chemokine ligand 5 [CCL 5], eotaxin), cytokines (tumor necrosis factor-alpha [TNF-α], IL-1α, IL-1β, IL-6, granulocyte-macrophage colony-stimulating factor [GM-CSF]), and pro-inflammatory mediators (nitric oxide [NO], inducible nitric oxide synthase [iNOS], cyclooxygenase-2 [COX2]) [9].

Lipopolysaccharide (LPS) is widely localized in the outer cell wall of Gram-negative bacteria. Thus, LPS elicits a host inflammatory response that results in increased production of chemokines, cytokines, and pro-inflammatory mediators by the immune system [10]. Macrophages exposed to LPS during microorganism infections stimulate the immune system to produce cytokines and chemokines, followed by inflammatory events. Thus, inhibiting macrophage activation by LPS is an important focus for therapeutic strategies aimed at treating inflammatory diseases [11,12,13,14].

Reactive oxygen species (ROS) are natural byproducts of oxygen metabolism that play important roles in cell signaling and homeostasis [15]. However, under various infection and pathological conditions, excess ROS production can cause protein and nucleic acid oxidation and can lead to toxic effects on cell structures [16]. Recent studies suggest that ROS can lead to activation of inflammatory effects via the mitogen-activated protein kinase (MAPK) signaling pathway, which induces the production of numerous inflammatory cytokines [17,18,19,20].

Sinapaldehyde (SNAH) is a methoxyphenol found in several plants such as *Senra incan* [21], *Ailanthus altissima* Swingle [22], *Eucalyptus globulus* [23], *Oryza sativa* [24], *Dendropanax dentiger* [25], and *Populus tomentosa* [26]. It was reported that SNAH exerts antibacterial activities against oral pathogenic bacteria, including *Streptococcus pyogenes*, *Streptococcus mitis*, and *Streptococcus mutans* [27]. Additionally, SNAH dose-dependently decreases ethyl phenylpropiolate-induced edema in the rat ear [21]. However, to our knowledge the anti-inflammatory effects of SNAH have not been examined; thus, the objective of this study was to investigate the anti-inflammatory activity of SNAH in macrophage RAW 264.7 cells. We investigated the effects of SNAH on cytokine (TNF-α and IL-6) production and its impact on the expression of COX-2 and iNOS proteins and TNF-α, iNOS, and IL-6 genes in LPS-induced RAW 264.7 cells. Furthermore, COX2 inhibitory effects of SNAH were analyzed using molecular docking simulation and enzyme inhibitory assays.

## 2. Results

### 2.1. Effect of SNAH on Cell Viability and LPS-Induced NO Production in RAW 264.7 Cells

**Figure 1:**
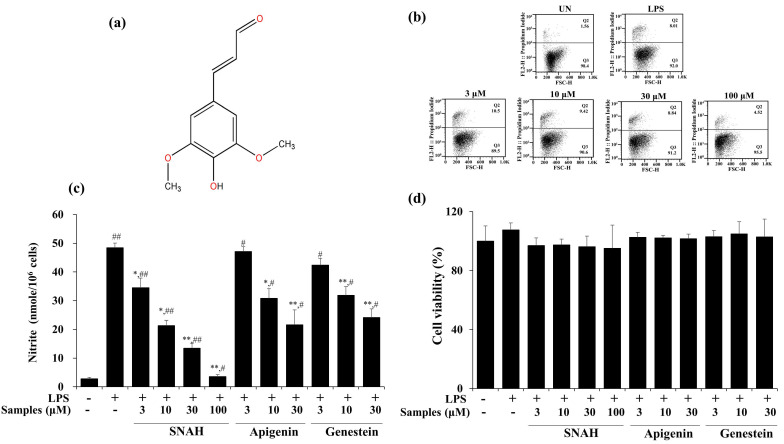
Effects of SNAH (sinapaldehyde) on cell viability and LPS (lipopolysaccharide)-induced NO (nitric oxide) production in RAW 264.7 cells. (**a**) The chemical structure of SNAH. (**b**) The viability of Raw264.7 cells was determined by FACS (fluorescence activated cell sorter) analysis. (**c**) Cells were pretreated with 3, 10, 30, and 100 μM SNAH for 1 h before treatment with 1 μg/mL LPS. After incubation for 18 h, NO production was detected by Griess test. (**d**) The cell viability was detected by XTT assay. The values are presented as means ± SD of three independent experiments. * *p* < 0.05, ** *p* < 0.01, compared to the LPS-treated group. # *p* < 0.05, ## *p* < 0.01, compared to the LPS non-treated group.

The cytotoxic effect of SNAH was examined using XTT (Cell Proliferation Kit II) assay. For SNAH concentrations ranging from 1–100 µM, cell viability was not significantly changed after 18-h treatment with 1 μg/mL LPS compared to control group cells. Flow cytometry is a sensitive method for detecting altered cell cytotoxicity. Propidium iodide penetrates the leaky membrane of dead/dying cells and binds DNA. Thus, it is widely used in flow cytometry assays [28]. Up to 100 μM, SNAH did not cause significant cytotoxicity (Figure 1d). Therefore, subsequent experiments were conducted at SNAH concentrations up to 100 µM. On the other hand, apigenin [29,30] and genistin [31], which are standard substances extracted from natural plants with anti-inflammatory activity, showed cytotoxicity at a concentration of 100 μM, and the experimental concentration was determined to be 30 μM. The effect of SNAH on NO production in LPS-induced RAW 267.4 cells was confirmed in a Griess reagent assay. LPS treatment significantly elevated (*p* < 0.05) the NO level by 18-fold. In contrast, treatment with 100 µM SNAH significantly inhibited NO production in RAW 264.7 cells stimulated with LPS. SNAH at 3, 10, 30, and 100 µM dramatically decreased NO formation by 29% ± 6.1%, 56% ± 2.8%, 72% ± 4.0%, and 93% ± 1.1%, respectively, compared to LPS-stimulated control cells (Figure 1b). However, apigenin and genistin decreased NO production by 55% ± 5.2% and 50% ± 3.0% activity, respectively, at 30 μM concentration (Figure 1c).

### 2.2. Effects of SNAH on Cytokine Production in RAW 264.7 Cells

**Figure 2:**
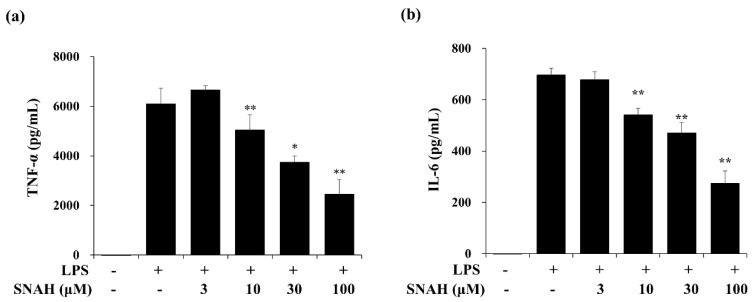
Effects of SNAH on LPS-induced TNF-α (**a**) and IL-6 (**b**) production. RAW 264.7 cells were pretreated with 3, 10, 30, and 100 μM SNAH for 1 h before treatment with 1 μg/mL LPS. After incubation for 18 h, IL-6 and TNF-α production was detected by ELISA. The values are presented as means ± SD of three independent experiments. * *p* < 0.05, ** *p* < 0.01 compared to the LPS-treated group.

Next, we measured the effect of SNAH on the expression of IL-6 and TNF-α in the supernatant of LPS-treated RAW 264.7 cells via enzyme-linked immunosorbent assay (ELISA). In cultures of LPS-treated cells, IL-6 and TNF-α concentrations were significantly increased. The LPS-induced increases were dose-dependently reversed by SNAH treatment. As shown in Figure 2, 30–100 μM SNAH significantly suppressed the production of IL-6 and TNF-α. The release of TNF-α and IL-6 was reduced by up to 59% ± 1.1% and 64% ± 3.5%, respectively, compared to LPS-stimulated control cells.

### 2.3. Effects of SNAH on iNOS and COX-2 Expression in RAW 264.7 Cells

**Figure 3:**
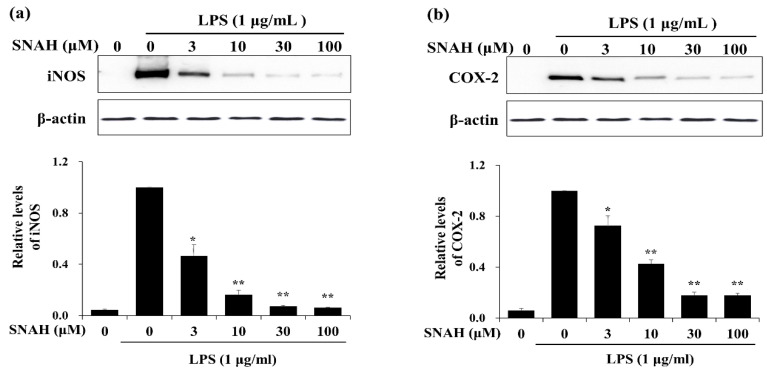
Effects of SNAH on LPS-induced iNOS and COX-2 protein expression. RAW 264.7 Cells were pretreated with 3-100 μM SNAH for 1 h before treatment with 1 μg/mL LPS. After incubation for 24 h, iNOS (**a**) and COX-2 (**b**) protein levels were detected by Western blot. The values are presented as means ± SD of three independent experiments. * *p* < 0.05, ** *p* < 0.01 compared to the LPS-treated group.

We examined the effects of SNAH on the expression of inflammatory factors in LPS-stimulated RAW 264.7 cells. Western blot analysis showed that 100 μM SNAH treatment decreased iNOS and COX-2 protein expression by 94% ± 1.2% and 82% ± 3.2%, respectively, compared to the LPS-stimulated control cells (Figure 3).

### 2.4. Effects of SNAH on Inflammatory Gene Expression in RAW 264.7 Cells

**Figure 4:**
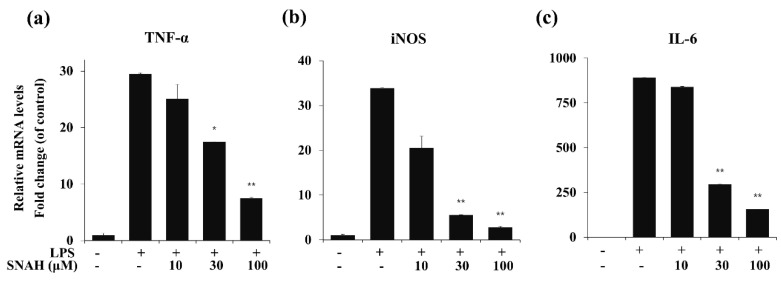
Effect of SNAH on TNF-α, iNOS, and IL-6 gene expression in RAW 264.7 cells. RAW264.7 cells were pretreated with 10-100 μM SNAH for 1 h prior to LPS stimulation. After 6h LPS stimulation, total RNA was isolated using Trizol reagent. TNF-α (**a**), iNOS (**b**), and IL-6 (**c**) mRNA levels were analyzed by real-time RT-PCR. Relative mRNA levels were determined using the Ct-value method and normalized to GAPDH expression. The values are presented as means ± SD of three independent experiments. * *p* < 0.05, ** *p* < 0.01 compared to the LPS-treated group.

To determine the effects of SNAH on LPS-induced expression of pro-inflammatory cytokines, mRNA levels of TNF-α, IL-6, and iNOS in RAW 264.7 cells were measured by real-time RT-PCR. As shown in Figure 4, LPS stimulation significantly increased the expression of TNF-α, IL-6, and iNOS mRNA compared to the control group in the absence of LPS. In contrast, IL-6, TNF-α, and iNOS mRNA were significantly (*p* < 0.01) downregulated by SNAH treatment. At 10, 30, and 100 μM, SNAH significantly (*p* < 0.01) inhibited iNOS mRNA expression by 39% ± 2.1%, 83% ± 1.8% and 92% ± 0.3% (*n* = 5), respectively, compared to LPS-stimulated control cells. The mRNA levels of pro-inflammatory cytokines IL-6 and TNF-α significantly (*p* < 0.01) decreased 74% ± 2.7% and 82% ± 3.6% (*n* = 5), respectively, compared to LPS-stimulated control cells.

### 2.5. Effect of SNAH on ROS Production in RAW 264.7 Cells and Radical Scavenging Activity

**Figure 5:**
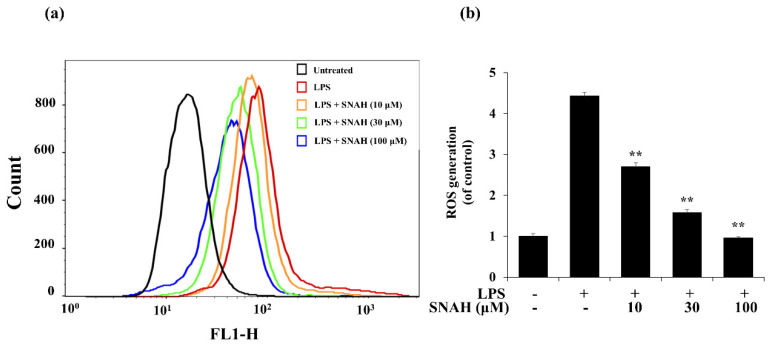
Effects of SNAH on LPS-induced ROS (reactive oxygen species) production. (**a**) RAW 264.7 cells were pretreated with 10–100 μM SNAH for 1 h before treatment with 1 μg/mL LPS for 18 h. Cells were incubated with 10 μM 2′,7′-dichlorofluorescein diacetate (DCFH-DA) for 30 min at 37 °C. Then, cells were harvested, and dichlorofluorescein (DCF) fluorescence was immediately analyzed by flow cytometry. DCF fluorescence intensity was determined from the same numbers of cells in a randomly selected area. (**b**) The mean relative ROS level is presented in a bar graph. The values are presented as means ± SD of three independent experiments ** *p* < 0.01, compared to the LPS-treated group.

Next, we investigated the effect of SNAH on ROS formation in LPS-treated RAW 264.7 macrophages. LPS treatment elevated intracellular ROS levels in macrophages after 24 h. In contrast, SNAH treatment significantly reduced LPS-induced ROS production in a dose-dependent manner. Treatment with 10, 30, and 100 µM SNAH significantly (*p* < 0.01) decreased ROS generation by 39% ± 3.1%, 64% ± 9.8%, and 78% ± 2.1%, respectively, compared to LPS-stimulated control cells (Figure 5). These findings suggest that the anti-inflammatory effect of SNAH is associated with its antioxidant effects in RAW 264.7 cells. To check the radical scavenging activity of SNAH, the DPPH radical-scavenging activity of the SNAH was carried out using ascorbic acid as a reference compound. At a concentration of 250 µM, SNAH identified radical scavenging effects of 73% ± 2% compared with 72% ± 2% for ascorbic acid. The amount of the SNAH necessary to decrease the DPPH radical by 50% (IC_50_) was calculated, and it was found that SNAH confirmed activity with a radical-scavenging effect of 172 µM compared with ascorbic acid (IC_50_ of 192 µM) (Table 1). Therefore, the ability of SNAH to strongly suppress ROS in LPS-stimulated RAW 264.7 macrophages might be associated with its ability to scavenge free radicals.

### 2.6. Effect of SNAH on MAPK and p65 Signaling in RAW 264.7 Cells

**Figure 6:**
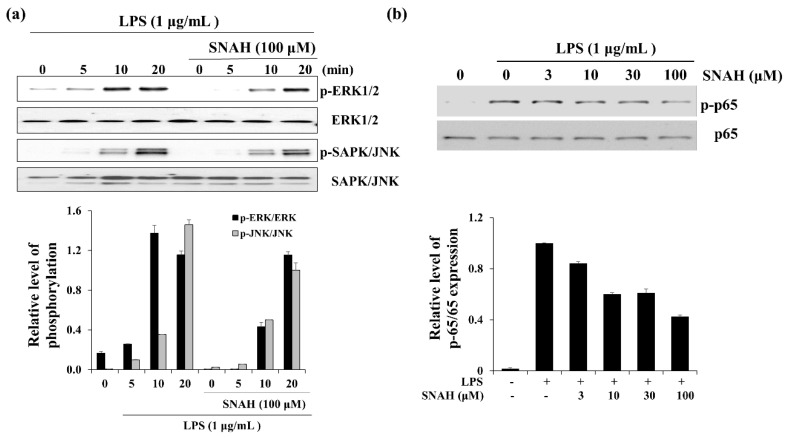
Effect of SNAH on MAPK and p65 signaling pathways. (**a**) The time course of ERK (extracellular-signal-regulated kinase) and SAPK/JNK (Stress-activated protein kinase/Jun-amino-terminal kinase) phosphorylation stimulated by LPS in RAW264.7 cells. (**b**) RAW 264.7 cells were treated for 15 min with LPS (1 μg/mL) alone or with LPS (1 μg/mL) coupled with 3–100 μM SNAH. Cell lysates were prepared and blotted with the indicated anti-phospho-antibodies. Total ERK1/2, SAPK/JNK, and NF-κB (p65) were probed as quantitative controls.

When RAW 264.7 cells were exposed to LPS, the phosphorylated ERK and SAPK/JNK (stress-activated protein kinase/Jun-amino-terminal kinase) increased in a time-dependent manner (Figure 6). Phosphorylated ERK1/2 was decreased in the presence of SNAH at 10 min. Moreover, SNAH treatment decreased SAPK/JNK phosphorylation after 20 min. In addition, phosphorylated p65 was induced in the LPS-stimulated group, while *p*-p65 decreased in the SNAH-treated group. At 10, 30, and 100 μM, SNAH inhibited p65 phosphorylation by 40%, 39%, and 57%, respectively, compared to LPS-stimulated control cells.

### 2.7. Molecular Docking Studies and COX-2 Enzyme Inhibitory Activity

**Figure 7:**
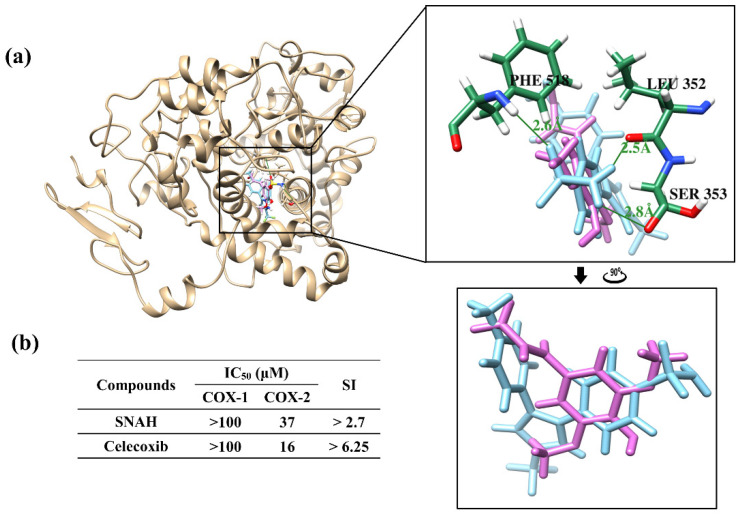
Docking simulation between SNAH and COX-2 (6COX). (**a**) The pink-colored chemical structure represents SNAH, and the COX-2 protein structure complexed with celecoxib is indicated by blue colored. The magnified rectangles indicate the active sites of COX-2 proteins. In the box image, hydrogen bond interactions between COX-2 and SNAH are depicted by green-colored lines. The binding energies of SNAH and celecoxib for COX-2 were −6.4 kcal/mol and −11.1 kcal/mol, respectively. (**b**) COX-2 enzyme inhibitory activities of SNAH. The commercial standard, celecoxib was used as a positive control at identical concentrations.

Docking simulations showed that SNAH was located properly at binding sites of COX-2. The docking score of SNAH for COX-2 was −6.4 kcal/mol, as measured by AutoDock Vina (Figure 7a). Celecoxib was used as positive control at the same COX-2 binding sites. The docking score of celecoxib was −11.1 kcal/mol. From the docking results, SNAH overlapped with the 4-methylpenyl ring of celecoxib in the binding pocket (Figure 7a). In addition, celecoxib had three hydrogen bond interactions with Leu352, Ser353, and Phe518 of COX-2. However, there were no hydrogen bond interactions between COX-2 and SNAH (Figure 7a).

The next study analyzed COX-2 enzyme inhibition activity of SNAH using COX-2 inhibitory assays. SNAH displayed selective inhibitors to COX-2 with IC_50_ values of 37 μM. The selectivity index (SI value) was calculated as the ratio of concentration (IC_50_) that inhibits the COX1 and COX2 isoforms [32]. SNAH and celecoxib displayed SI values of >2.7 and >6.25, respectively (Figure 7b). The results of COX inhibition assay indicated that SNAH had a reasonable inhibitory activity against COX-2. Further, SNAH showed no inhibition for COX-1 up to 100 μM. Therefore, our results indicate that SNAH interacts with COX-2 and is a potential COX-2 inhibitor.

## 3. Discussion

The progression of inflammation is the main cause of many disease conditions, including headache, atopy, metabolic syndrome, multiple sclerosis, Alzheimer’s disease, and Parkinson’s disease [33,34,35]. When cells are infected by Gram-negative bacteria, LPS in the outer membrane of Gram-negative bacteria activates macrophages, thereby causing them to secrete high concentrations of pro-inflammatory cytokines, and inflammatory factors or mediators. Therefore, LPS-induced inflammatory effects in macrophages are widely utilized to screen anti-inflammatory agents [36]. Macrophage stimulation by LPS promotes excessive NO release via degradation of L-arginine. When responding to inflammatory stimuli in the mammalian immune system, NO generation is regulated by iNOS in macrophage cells [37]. In addition, LPS-induced macrophages produce excess inflammatory mediators, including COX2, a key enzyme in PGE_2_ synthesis, and inflammatory cytokines such as TNF-α and IL-6 [38]. There are data changes at each time point of the LPS-induced inflammatory mediator [39]. Firstly, the stimulus of LPS caused the immediate release of TNF-α until 12 h, and then IL-6 was reached a peak at 18 h. The iNOS expression showed a similar change comparing with IL-6. The change of NO release was influenced by the iNOS contents [40]. Therefore, they are the targets of anti-inflammatory reagents in this study. SNAH reduced the production of NO, TNF-α, and IL-6 in LPS-stimulated RAW 264.7 cells in a dose-dependent manner (Figure 2). Moreover, NO inhibitory activity of SNAH was higher than that of apigenin and genistein, standard substances, under the same conditions (Figure 1c).

Nitric oxide synthases (NOSs) exist in three isoforms: endothelial NOS (eNOS), neuronal NOS (nNOS), and iNOS [41]. eNOS and nNOS are constitutively expressed, while iNOS is expressed in response to inflammatory stimuli [42]. iNOS is especially activated in response to inflammatory stimuli such as cytokines, interleukins, and bacterial endotoxin [43]. Cyclooxygenase (COX) exists in two isoforms: COX-1 and COX-2. COX-1 is a constitutively expressed enzyme with general housekeeping functions [44]. COX-2 is an inducible enzyme that catalyzes the biosynthesis of PGE2, which contributes to the pathogenesis of various inflammatory diseases. PGE2 synthesis is catalyzed by the COX enzyme which is expressed during all inflammatory processes [45]. In inflammatory cells including macrophages, COX-2 and iNOS are induced by inflammatory stimuli such as LPS, resulting in the aggravated cellular inflammatory signaling. iNOS and COX-2 are key enzymes that are involved in NO and PGE2 synthesis, respectively. Therefore, they are the major targets of anti-inflammatory agents in current research [46,47,48]. Our results showed that SNAH exerts its anti-inflammatory effects by inhibiting iNOS and COX-2 protein expression (Figure 3). Additionally, SNAH regulates iNOS expression at both the protein and gene levels (Figure 4). These findings indicate that SNAH may effectively attenuate inflammatory responses by decreasing NO production via the inhibition of iNOS and COX-2 protein expression, mRNA levels, and pro-inflammatory cytokines secretion.

ROS accumulation from the endogenous antioxidant reaction results in a redox imbalance that leads to oxidative stress [49]. Increased ROS production is strongly associated with many other pathological conditions, such as inflammation. Inflammatory mediators and cytokines promote an influx of macrophages that, in turn, accelerate intracellular ROS accumulation [50]. In addition, ROS act as secondary messengers and participate in the inflammatory signaling pathway [51]. Therefore, the ability of SNAH to strongly suppress ROS in LPS-stimulated RAW 264.7 macrophages might be attributed to its ability to scavenge free radicals (Table 1). SNAH-mediated inhibition of ROS generation may also potentially inhibit the expression of pro-inflammatory mediators and cytokines, thereby explaining the strong anti-inflammatory properties of SNAH.

Many studies indicate that the toll-like receptor (TLR) family of pattern recognition receptors include central mediators of the inflammatory response. Accumulating evidence indicates that TLR4 specifically mediates LPS-induced signaling [52]. The first step in the LPS/TLR4-mediated inflammatory signaling pathway is the binding of LPS to TLR4 complexed with MYD88 adapters at the plasma membrane. This binding initiates intracellular signaling cascades. Activation of TLR4 by LPS induces PI3K/Akt, MAPK, and IB kinase complex phosphorylation, eventually resulting in the activation of NF-κB [53,54]. Further studies are necessary to elucidate the molecular mechanism by which SNAH exerts its inhibitory effects by evaluating the role of TLR-4/MAPK/NF-κB pathway activation.

Docking simulations showed that binding scores obtained from SNAH and celecoxib were correlated with IC_50_ values. Two compounds had similar binding orientations, with overlap between the dimethoxyphenyl ring of SNAH and the 4-methylpenyl ring of celecoxib in the same binding pocket. The pocket site forms an important hydrophobic cavity for the 4-methylpenyl ring of celecoxib to bind COX-2 [55]. Celecoxib forms hydrogen bonds with COX-2 at Leu352, Ser353, and Phe513. We suggest that those hydrogen bonds could increase the binding affinity with COX-2, and that the modification of the SNAH dimethoxyphenyl ring might improve COX-2 inhibition because the substitution of methoxy group at *para* position was responsible for its high potency against COX-2 and high selective index [56].

## 4. Materials and Methods

### 4.1. Materials

Dimethyl sulfoxide, apigenin, genistein, LPS (from an *Escherichia coli* strain), 2,7-dichlorofluorescein diacetate (DCF-DA), Griess reagent, and the Cell Proliferation Kit II (XTT) were obtained from Sigma (St. Louis, MO, USA). Dulbecco’s Modified Eagle’s Medium (DMEM), fetal bovine serum, penicillin (100 IU/mL)-streptomycin (100 μg/mL), and trypsin-EDTA were purchased from Gibco (Grand Island, NY, USA). The ELISA kits for TNF-α and IL-6 were purchased from R&D Systems, Inc. (Minneapolis, MN, USA). Antibodies against *p*-ERK1/2, ERK1/2, *p*-SAPK/JNK, SAPK/JNK, iNOS, COX2, and β-actin, and secondary antibodies were purchased from Cell Signaling Technology (Danvers, MA, USA). SNAH was obtained from ChemFaces Biochemical Co. Ltd. (Wuhan, China), and the purity of SNAH was ≥98%. It was dissolved at a concentration of 100 mM in dimethyl sulfoxide. The IUPAC nomenclature of SNAH is (*E*)-3-(4-hydroxy-3,5-dimethoxyphenyl)acrylaldehyde, and the chemical structure was drawn using ChemDraw software (https://www.perkinelmer.com/category/chemdraw).

### 4.2. Cell Culture

RAW 264.7 macrophages were obtained from the American Type Culture Collection (Manassas, VA, USA). The cells were grown at 37 °C in DMEM supplemented with 10% fetal bovine serum and penicillin sulfate in a humidified 5% CO_2_ atmosphere. The cells were pretreated with SNAH for 1 h, and then stimulated with 1 μg/mL LPS for the indicated time.

### 4.3. Cell Viability Assay

Cell viability studies were performed using the XTT assay. RAW 264.7 cells were plated at a density of 5 × 10^5^ cells/mL in a 96-well plate, pretreated with isolated SNAH for 1 h, and stimulated with 1 μg/mL LPS for 24 h. XTT assay was measured using a Cell Proliferation Assay Kit II (Roche, Basel, Switzerland). Briefly, the XTT labeling mixture was prepared by mixing 50 volumes 1 mg/mL sodium 3′-[1-(phenylaminocarbonyl)-3,4-tetrazolium]-bis(4-methoxy-6-nitro) benzene sulfonic acid hydrate (in media) with 1 volume 0.383 mg/mL *N*-methyldibenzopyrazine methyl sulfate in PBS. This mixture was added to the cultures and incubated for 1 h at 37 °C. Absorbance was measured at 490 nm with a reference wavelength at 650 nm. The cell viability results were presented as a ratio relative to untreated (UN) samples. For fluorescence-activated cell sorting analysis, the cells were washed in PBS and stained with 0.05 μg propidium iodide for 15 min at room temperature. Flow cytometry analysis was performed on a BD FACSCalibur (BD Biosciences, Franklin Lakes, NJ, USA) using FlowJo (TreeStar, Ashland, OR, USA) and Diva software. Viability was calculated by comparing treated cells to non-treated cells.

### 4.4. Measurement of Nitric Oxide Production

RAW 264.7 cells were seeded into 96 well plates at a density of 5 × 10^5^ cells/mL for 24 h. The medium was replaced with fresh medium containing 1 μg/mL LPS and 3–100 μM SNAH in the test group, followed by incubation for 24 h. Nitrite accumulated in the culture supernatant was measured as an indicator of NO production using Griess reagent. The culture supernatant was mixed with equal volumes Griess reagent (1% (*w*/*v*) sulfanilamide in 5% (*v*/*v*) phosphoric acid and 0.1% (*w*/*v*) naphthylethylenediamine-HCl) for 10 min. Absorbance at 540 nm was measured in a microplate spectrophotometer (Bio-Tek, Vermont, VT, USA). Fresh culture medium was used as the blank in all experiments. The amount of nitrite in the samples was determined by referring to a sodium nitrite standard curve.

### 4.5. Measurement of Cytokines by ELISA

The secretion of pro-inflammatory cytokines, including IL-6 and TNF-α, was measured with an ELISA assay kit (R&D Systems, Minneapolis, MN, USA). RAW 264.7 cells were seeded into a 96-well plate containing 3–100 μM SNAH with or without 0.2 μg/mL LPS for 6 h. The supernatant was harvested, and pro-inflammatory cytokine levels were determined using ELISA kits. Absorbance was measured at 450 nm using a microplate spectrophotometer (Bio-Tek). The quantities of cytokines secreted by RAW 264.7 cells were calculated using standard curves.

### 4.6. Western Blot Analysis

iNOS, COX-2, phospho-ERK1/2, SAPK/JNK, and actin protein expression was determined by Western blotting. RAW 264.7 cells were cultured in 6-well plates (6 × 10^5^ cells/well) with or without 3–100 μM SNAH and 1 μg/mL LPS for 24 h. Cells were lysed with RIPA (radioimmunoprecipitation assay) buffer [50 mM Tris-Cl (pH 8.0), 5 mM EDTA, 150 mM NaCl, 1% NP-40, 0.1% SDS and 1 mM phenylmethylsulfonyl fluoride]. Lysates were centrifuged at 12,000 rpm for 20 min. Supernatants were collected and protein concentrations were determined using a Bio-Rad Protein Assay kit (Bio-Rad Laboratories, Inc., Hercules, CA, USA). Equal amounts of proteins were resolved by 10% SDS-polyacrylamide gel electrophoresis and transferred to nitrocellulose membranes. The membranes were incubated with blocking buffer (Tris-buffered saline containing 0.2% Tween-20 and 5% non-fat dried milk) and probed with the indicated primary antibodies. After washing, membranes were probed with horseradish peroxidase-conjugated secondary antibodies. Detection was performed using an enhanced chemiluminescence protein detection system (Amersham Biosciences, Little Chalfont, UK). The chemiluminescent signal was detected by 10–120 s exposure using a ChemiDoc Touch Gel Imaging System (Cat. No. 1708370, Bio-Rad, Hercules, CA, USA). Western blotting analyses were repeated three times, and representative results are shown.

### 4.7. Measurement of Intracellular Reactive Oxygen Species Formation

Intracellular ROS levels were quantified using a ROS-sensitive fluorescence indicator, DCFH-DA. In viable cells, 2,7-dichlorofluorescein diacetate (DCFH-DA) is cleaved to DCFH by esterase, followed by ROS oxidation to form a highly fluorescent molecule, DCF. Briefly, RAW 264.7 cells were plated at a density of 5 × 10^5^ cells/mL in a 6-well plate, pretreated with isolated SNAH for 1 h, and stimulated with 1 μg/mL LPS for 24 h. Then, they were washed twice with 1 × washing buffer and loaded with 10 μM DCFH-DA detection reagent. After incubation in darkness for another 30 min at 37 °C, cells were washed twice with 1 × washing buffer. Fluorescence representing intracellular ROS levels was measured at 485/20 nm excitation and 528/20 nm emission in a fluorescence multi-detection reader (Glomax, Promega, Madison, WI, USA). FACS data were measured with a BD FACSCalibur (BD Biosciences, San Jose, CA, USA) at the excitation wavelength of 488 nm and the emission wavelength of 525 nm. Data were analyzed by FlowJo software (Tree Star, San Carlos, CA, USA).

### 4.8. DPPH Radical Scavenging Assay

The DPPH radical scavenging assay was conducted using the published protocol method [57]. Briefly, the compound (100 μL) was added to 100 μL of 2 mM DPPH solution in 50% ethanol. The mixtures were mixed well and incubated in the dark for 30 min. The reduction of DPPH absorption was measured at 520 nm using a microplate spectrophotometer (Bio-Tek). Ascorbic acid (100 μM) was used as a positive control. All determinations were performed in triplicate. The DPPH radical scavenging activity was calculated using Equation (1):

DPPH scavenging (%) = [1 − (Absorbance of the sample/Absorbance of the control)] × 100(1)

### 4.9. Real-Time Reverse Transcription-Polymerase Chain Reaction (RT-PCR) Analysis

Total RNA was extracted using an RNeasy Mini Kit (QIAGEN, Valencia, CA, USA) and cDNA was synthesized using AccuPower RT PreMix (BIONEER Corporation, Daejeon, Korea). SYBR Green PCR Master Mix (Life Technologies Corporation, Carlsbad, CA, USA) and an ABI 7500 Sequence Detection System (Applied Biosciences, Foster City, CA, USA) were used for real-time PCR analysis. Primer sequences used in this study are listed in Table 1. Samples were amplified by 40 cycles of 15 s at 95 °C and 1 min at 60 °C. All reactions were run in triplicate, and relative expression levels were determined with the 2^−∆∆CT^ method by normalizing the expression levels to GAPDH. The primer list is in Table 2.

### 4.10. COX-1/2 Inhibition Assay

The inhibitory activity of each COX-1/2 ligand was detected using a COX-1/2 Inhibitor Screening Kit (BioVison, Milpitas, CA, USA). COX Assay Buffer (75 μL), COX Cofactor working solution (2 μL), COX probe solution (1 μL), recombinant COX-1 or COX-2 (1 μL), arachidonic acid/NAOH solution (10 μL), and test solution (10 μL) were mixed in a 96-well plate, and fluorescence kinetics were measured for 10 min at 25 °C. The fluorescence value of each well was measured with an excitation wavelength of 535 nm and an emission wavelength of 587 nm. Celecoxib, a COX-2 inhibitor, was used as the positive control group. Two points (T_1_ and T_2_) in the linear range of the plot were used to obtain the corresponding fluorescence values (RFU_1_ and RFU_2_). Then, the slope for all samples (S) was calculated by dividing the net ΔRFU (RFU_2_ − RFU_1_) values by the time ΔT (T_2_ − T_1_) as follows in Equation (2):

Relative inhibition (%) = (slope of the Enzyme Control − slope of S)/(slope of Enzyme Control) × 100(2)

The concentration of the test compound causing 50% inhibition (IC_50_, μM) was calculated from the concentration inhibition response curve. The selectivity indices (SI, COX-1 IC_50_/COX-2 IC_50_) were also calculated and compared with that of the standard COX-2 selective inhibitor, celecoxib.

### 4.11. Molecular Docking

The AutoDock Vina program [58] was used to simulate COX-2 and SNAH docking, and celecoxib as a positive control. The docking pocket was previously identified in active sites of COX-2 complexes (PDB ID: 6COX). To prepare the docking simulation, the 3D structures of SNAH (Pubchem CID: 5280802) and celecoxib (Pubchem CID: 2662) were downloaded from PubChem (https://pubchem.ncbi.nlm.nih.gov/). Images of complexes and hydrogen bond predictions were obtained using Chimera version 1.14 (https://www.cgl.ucsf.edu/chimera/).

### 4.12. Statistical Analysis

Data are expressed as the mean ± standard deviation from three independent experiments. Statistical significance was determined by Student’s t-test for independent means using Microsoft Excel. Data are presented as the mean ± SD of three independent experiments; *p* < 0.05 was considered statistically significant.

## 5. Conclusions

The results presented here demonstrate that SNAH exerted potent anti-inflammatory effects on RAW 264.7 macrophages. Treatment of LPS-stimulated RAW 264.7 macrophages with SNAH significantly attenuated the production of NO, TNF-α, and IL-6 by reducing their corresponding gene expression. The SNAH as a natural product had meaningful IC_50_ value to inhibit COX-2 and possible mode of action in the same pocket with celecoxib. The ability of SNAH to inhibit the inflammatory response was associated with a reduction of intracellular ROS production. The results of this study support the use of SNAH as an alternative candidate for safe and effective treatment of inflammatory diseases. Further studies are needed to understand the precise molecular mechanisms regulating the anti-inflammatory activity in the animal model and validate it as a modulator of macrophage activation.

## Figures and Tables

**Table 1:** DPPH radical-scavenging activity of the SNAH and IC_50_ values.

Compounds	DPPH ^1^ Radical Scavenging Activity (%)				IC_50_ (μM)
	62.5 μM	125 μM	250 μM	500 μM	
SNAH	25% ± 5% ^2^	48% ± 2%	73% ± 2%	87% ± 1%	172
Ascorbic acid	19% ± 1%	49% ± 5%	72% ± 2%	84% ± 3%	192

^1^ 2,2′-diphenyl-1-picrylhydrazyl; ^2^ Values are expressed as mean ± SD of triplicate determinations.

**Table 2:** List of primers used in this study.

Gene	Primer Sequence	Accession No
mouse iNOS	Forward 5′-GCATCCCTGTGGAGGACAACC-3′	M20234
	Reverse 5′-GCATCCCTGTGGAGGACAACC-3′	
mouse TNF-α	Forward 5′-GCATCCCTGTGGAGGACAACC-3′	BC076598
	Reverse 5′-AAGACGCTGCACTGCTGGTC-3′	
mouse IL-6	Forward 5′-TCTGGCCTCCAGTTACCAAC-3′	EU554632
	Reverse 5′-TCAGTGAGGAGAGGCTGGTT-3′	
mouse GAPDH	Forward 5′-GCGAGACCCCACTAACATCA-3′	GU214026
	Reverse 5′-GAGTTGGGATAGGGCCTCTCTT-3′	

## Abbreviations

LPS	Lipopolysaccharide
NO	Nitric oxide
ROS	Reactive oxygen species
DPPH	2,2-diphenyl-1- picrylhydrazyl
TNF	Tumor necrosis factor
iNOS	Inducible nitric oxide synthase
eNOS	Endothelial NOS
nNOS	Neuronal NOS
COX-2	Cyclooxygenase-2
SI	Selectivity indices
ELISA	Enzyme-linked immunosorbent assay
TLR	Toll-like receptor
DCF-DA	2,7-dichlorofluorescein diacetate
DMEM	Dulbecco’s Modified Eagle’s Medium
SAPK/JNK	Stress-activated protein kinase/Jun-amino-terminal kinase
RIPA	Radioimmunoprecipitation assay
